# Multiple Ruptured Aneurysms Over Basilar Artery Fenestration: Endovascular Management

**DOI:** 10.7759/cureus.21719

**Published:** 2022-01-29

**Authors:** Sibasankar Dalai, Madhusudhana Babu Marthati, Aravind Varma Datla, Mohan V. Sumedha Maturu, Ramu Naidu Yellapu

**Affiliations:** 1 Interventional Neuroradiology, Medicover Hospitals, Visakhapatnam, IND; 2 Neurology, Apollo Hospitals, Visakhapatnam, IND; 3 Internal Medicine, Medicover Hospitals, Visakhapatnam, IND; 4 Neurology, Medicover Hospitals, Visakhapatnam, IND; 5 Critical Care Medicine, Medicover Hospitals, Visakhapatnam, IND

**Keywords:** ct angiography, mr angiography, aneurysmal subarachnoid hemorrhage, cerebral digital substraction angiography, endovascular interventions, flow diversion, aneurysm coiling, aneurysmal rupture, aneurysm, basilar artery fenestration

## Abstract

Basilar artery fenestrations (BAF) are rare vascular anomalies. Surgical intervention for aneurysms in this vascular segment is exceptionally arduous because of the complexity of the neurovascular structures in the vicinity of the brainstem. Endovascular therapy (ET) is the treatment of choice because of its safety and efficacy. Here, we report a 62-year-old female presenting with a two-day history of sudden onset severe headache, vomiting, and altered sensorium. A computed tomography (CT) and subsequent CT angiogram (CTA) revealed subarachnoid hemorrhage (SAH) and BAF with an aneurysm on each arm of the fenestration. Digital subtraction angiogram (DSA) with a three-dimensional rotational angiogram (3DRA) was employed before initiating ET. We used coiling and flow diversion to exclude the aneurysms from circulation. A six-month follow-up angiography reconfirmed the complete obliteration of the aneurysms. There was no focal neurological deficit.

## Introduction

Basilar artery fenestration (BAF) is a rare intracranial vascular anomaly and is related to embryogenesis. The reported incidence varies from 0.6% to 2.33% depending on the diagnostic modality [1-3]. Although challenging, an endovascular approach is the treatment of choice, owing to the complex neurovascular anatomy in the vicinity of the brainstem [4]. BAF is typically located near the vertebrobasilar artery junction. Fenestration of the middle and distal segments is a rare occurrence. Currently, the literature regarding BAF is sparse and is confined mainly to case reports and series [5].

We report a unique case of two small aneurysms, one in each arm of the fenestration. An aneurysmal bleed occurred, leading to a subarachnoid hemorrhage (SAH). Due to the close anatomical proximity of the aneurysms, it was difficult to pinpoint the origin of the bleed. Both aneurysms were managed successfully by coiling the aneurysm in the left arm and flow diversion in the right arm without any complications and an excellent clinical outcome. At the six-month follow-up, the clinical examination revealed no focal neurological deficit, and cross-sectional imaging showed complete exclusion of the aneurysms from the circulation.

## Case presentation

**Figure 1:**
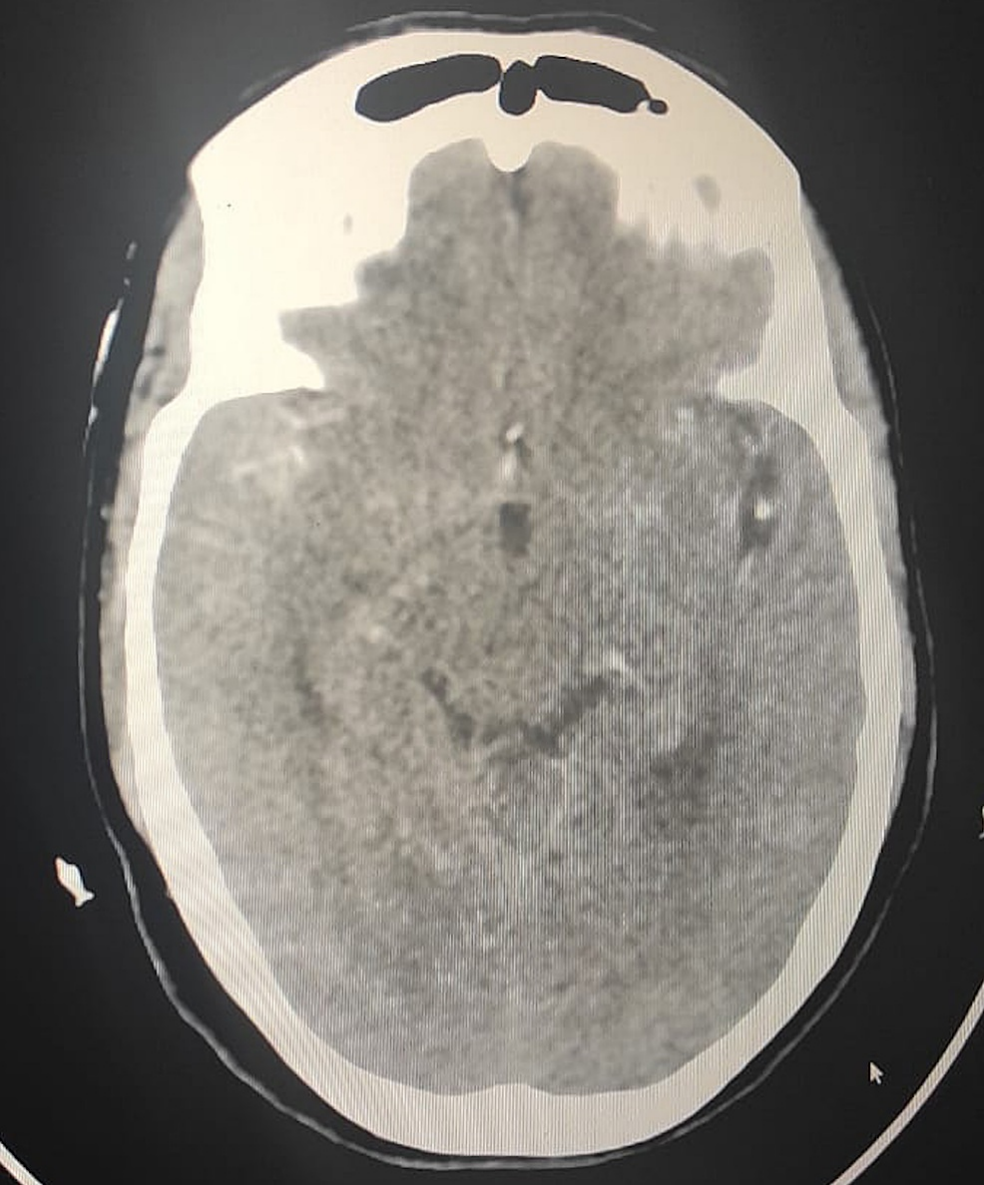
Plain CT scan of the brain showing a subarachnoid hemorrhage CT, Computed tomography.

A 62-year-old female patient presented to our hospital with a two-day history of sudden onset severe headache. She is a known hypertensive on antihypertensive treatment with good compliance. There was no past medical history of diabetes, smoking, or alcohol consumption. There was no antecedent trauma or fall. On the second day of her symptoms, she developed vomiting, gradually became drowsy, and was rushed to our emergency department. Glasgow coma scale (GCS) at presentation was 8/15. Blood pressure was 168/90 mmHg, and heart rate was 59 bpm. Her physical examination revealed neck rigidity and positive Kernig's sign. A plain CT scan of the brain revealed a subarachnoid hemorrhage (Figure 1).

**Figure 2:**
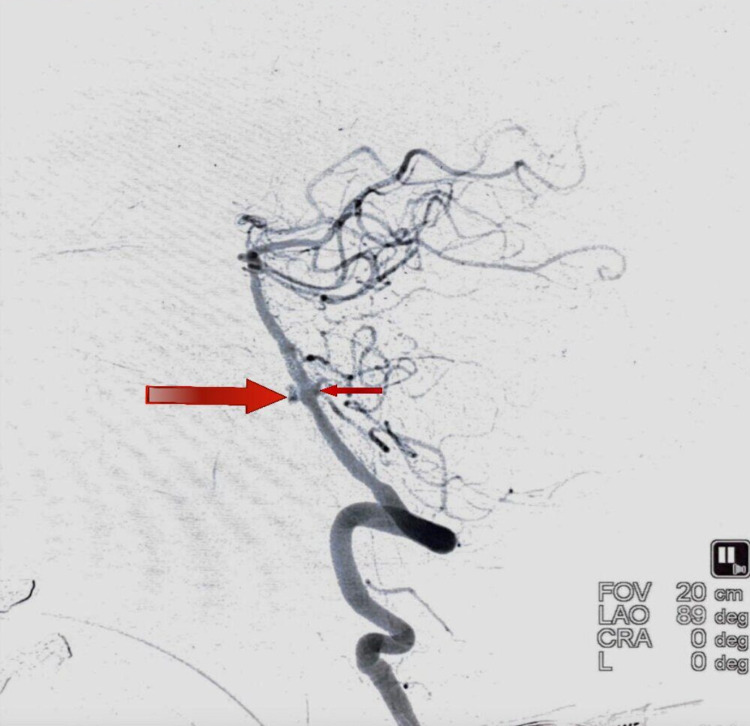
DSA lateral view demonstrating an aneurysm directed anteriorly (larger arrow) and another aneurysm directed posteriorly (smaller arrow) DSA, Digital subtraction angiogram.

A subsequent CT angiogram of the brain revealed a fenestration abnormality of the basilar artery at the proximal segment near the vertebrobasilar junction. Each arm of the fenestration revealed a tiny aneurysm, measuring 2.5 mm on the right arm and 3.5 mm on the left arm (Figure 2).

**Figure 3:**
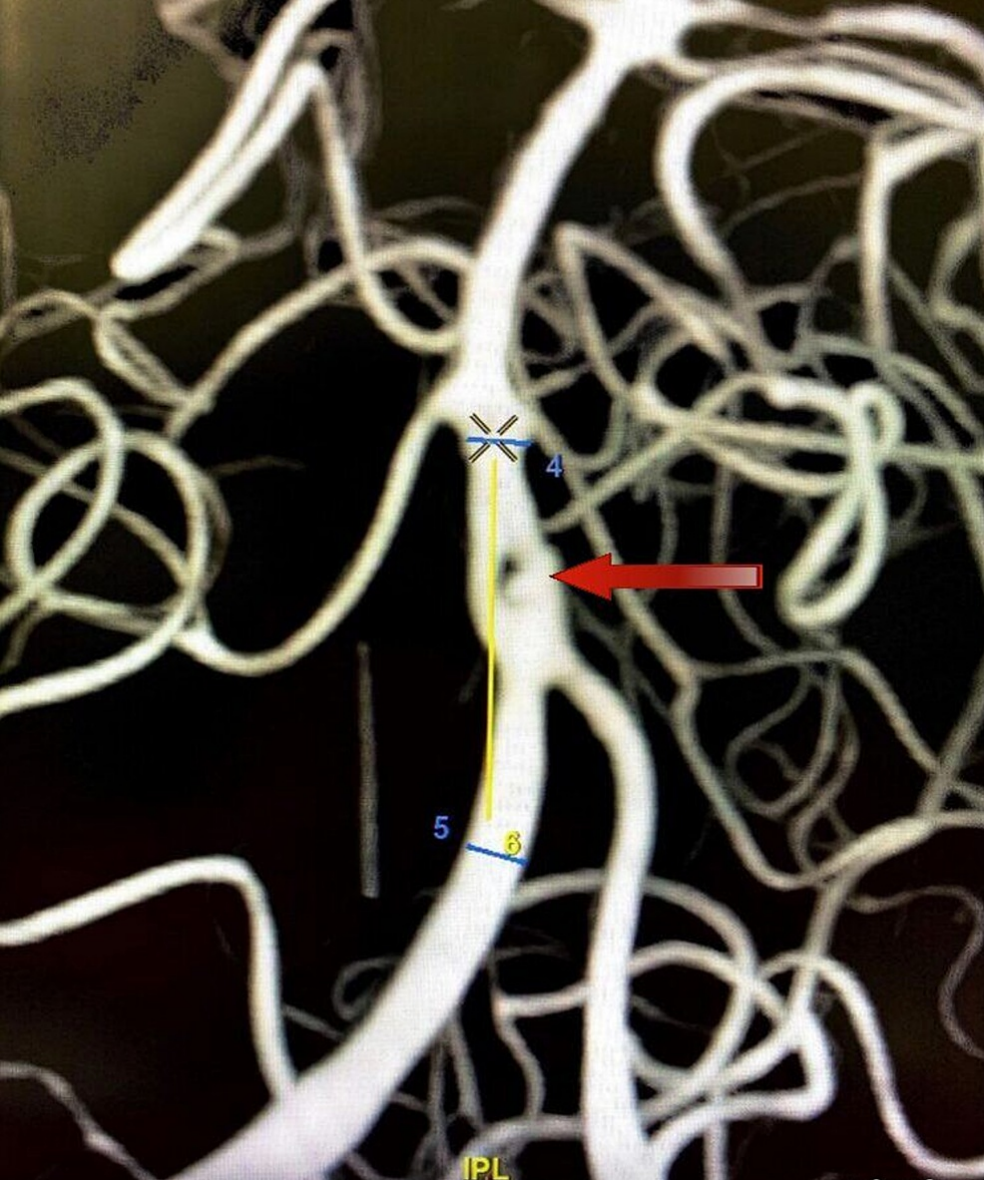
DSA-3DRA anterior view showing the anteriorly directed, larger narrow-necked left-arm aneurysm DSA, Digital subtraction angiogram; 3DRA, three-dimensional rotational angiogram.

A DSA with 3DRA (Figure 3) of the head and neck vessels was done to visualize this complex presentation better and to aid in the decision-making process.

**Figure 4:**
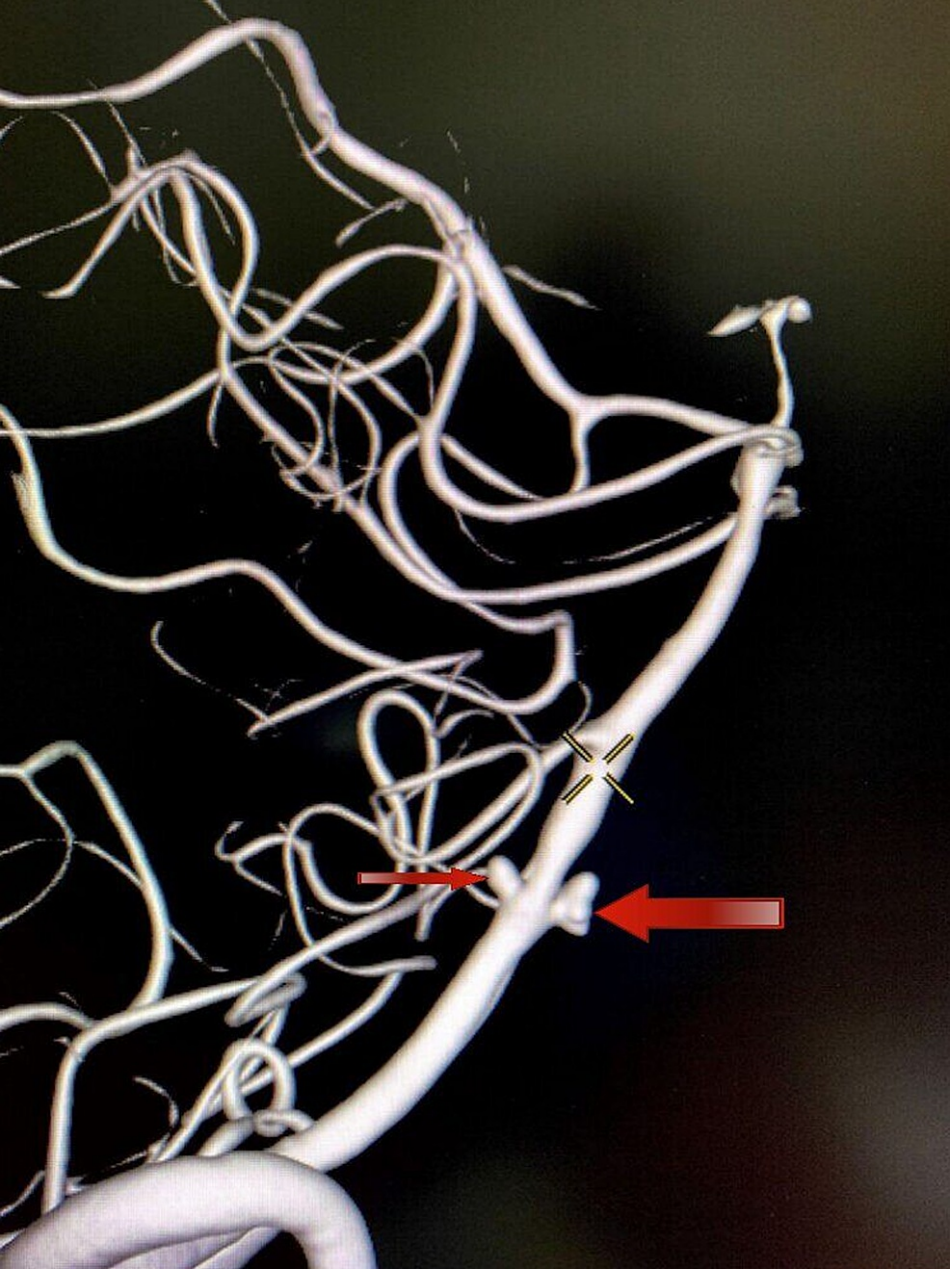
DSA-3DRA lateral view demonstrating two aneurysms. The left-arm aneurysm (larger arrow) was directed anteriorly. The right aneurysm (smaller arrow) was directed posteriorly. DSA, Digital subtraction angiogram; 3DRA, three-dimensional rotational angiogram.

The right aneurysm was directed posteriorly, whereas the left aneurysm was directed anteriorly (Figure 4). So, a decision was taken to coil the larger, narrow-necked left aneurysm and achieve flow diversion in the smaller, blister-like wide-necked right aneurysm.

Procedure

**Figure 5:**
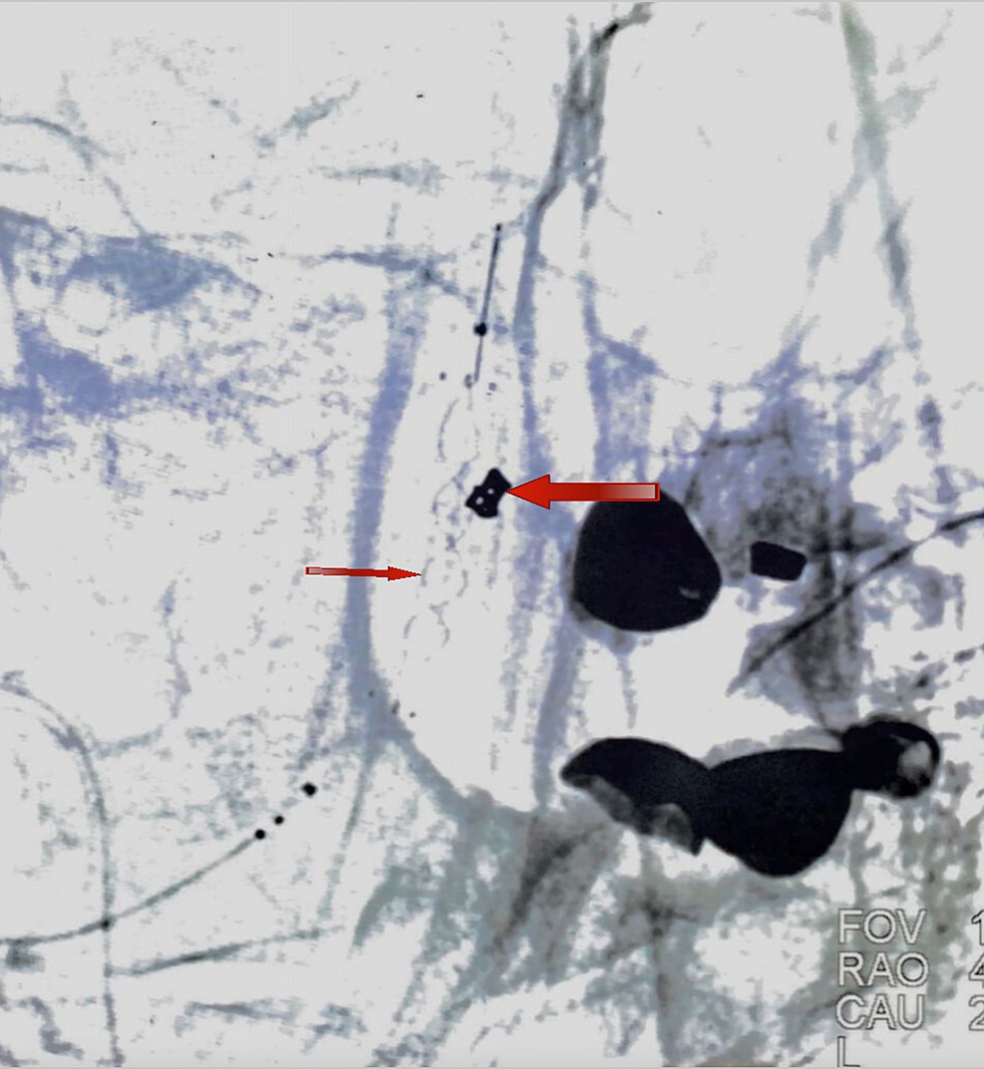
DSA anterior view showing a left coiled aneurysmal sac (larger arrow) and LVIS stent in the right arm of the fenestration DSA, Digital subtraction angiogram; LVIS, low-profile visible intraluminal support.

The patient was placed supine on the angiographic table. The patient was intubated, and the procedure was performed under general anesthesia. A right transfemoral arterial approach was taken. A 6F short sheath was placed into the common femoral artery. The left vertebral artery was accessed with a 5F vertebral artery catheter. The vertebral artery catheter was exchanged for a 6F Ballast 088 long sheath (BALT USA, Irvine, CA). The Ballast was placed at the distal V2 segment of the vertebral artery. A 6.3F DAC 070 (distal-access catheter; Concentric Medical, Mountain View, CA) was taken inside the Ballast and placed in the V4 segment of the left vertebral artery. An SL-10 Excelsior microcatheter (Stryker Neurovascular, CA) and Synchro 0.014 wire (Stryker Neurovascular, CA) were used, and the left aneurysm with a maximum diameter of 3.5 mm was canulated. A 3 x 10 coil was placed under continuous road-map guidance. The aneurysm could be entirely excluded from the circulation after the coiling (Figure 5).

**Figure 6:**
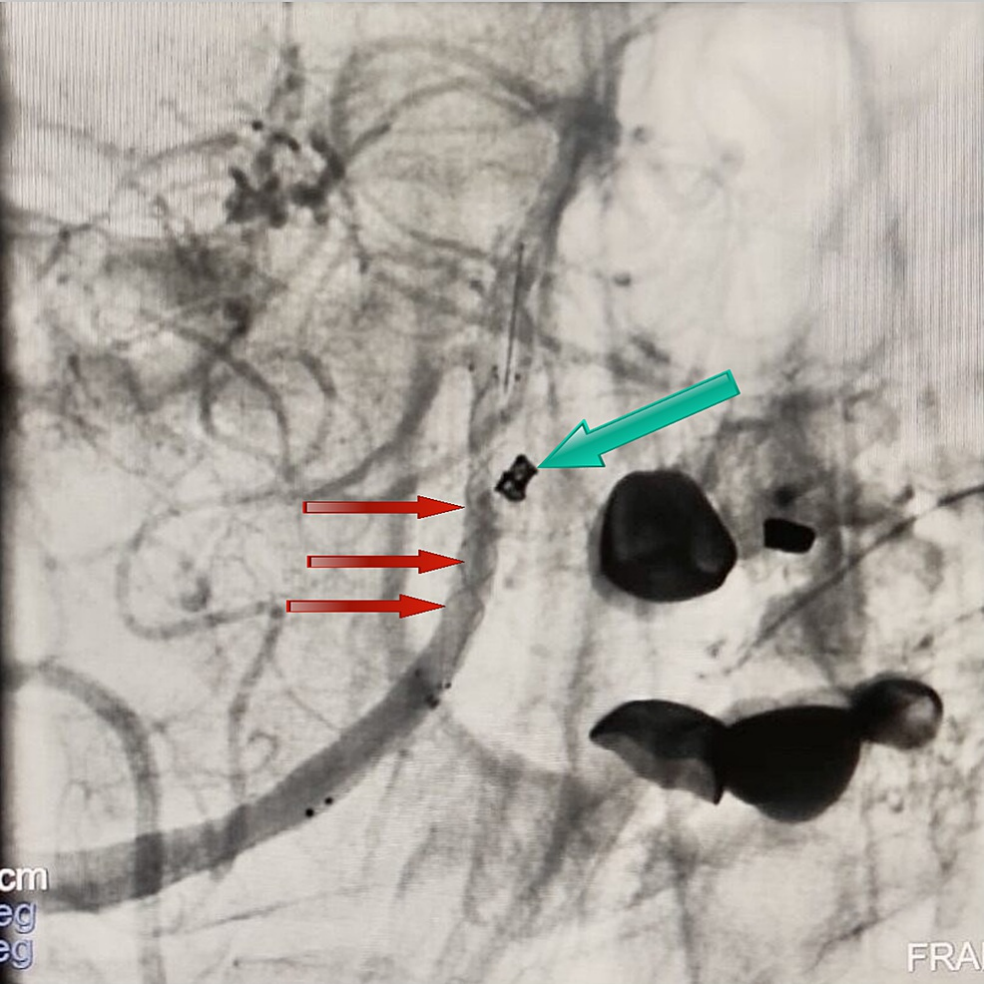
DSA anterior view showing the radio-opaque margin of the stent used to achieve flow diversion within the right arm of the fenestration (red arrows) and the placement of coils within the left aneurysmal sac (green arrows) DSA, Digital subtraction angiogram.

The aneurysm of the right fenestrated arm was 2.5 mm in its largest diameter with a wide neck. A Headway 21 microcatheter (Microvention, Aliso Viejo, CA) with a Synchro 0.014 wire was used, and the aneurysm was crossed. The catheter was placed in the mid-basilar artery. A 3.5 mm x 23 mm low-profile visible intraluminal support (LVIS) (Microvention, Aliso Viejo, CA) was navigated in the Headway microcatheter. The LVIS stent was deployed across the right limb of the BAF. The distal end of the LVIS was placed just below the origin of the anterior inferior cerebellar artery, and adequate coverage of the aneurysm was achieved (Figure 6).

**Figure 7:**
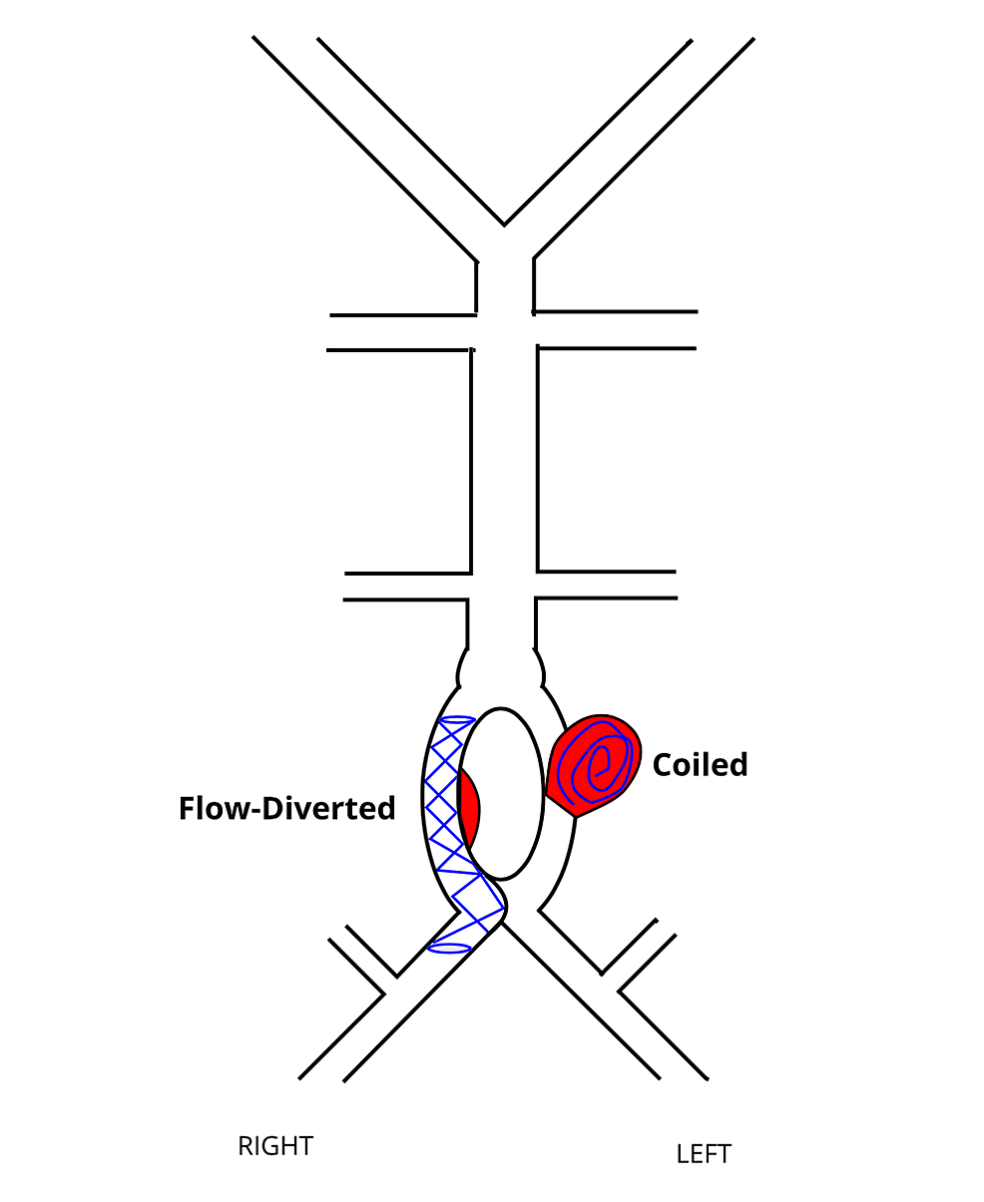
A schematic showing flow-diverted right arm of the fenestration and coiled left-arm aneurysmal sac

The final angiogram demonstrated stagnancy of flow within the right aneurysm. The procedure was completed, and the patient was extubated successfully (Figure 7).

**Figure 8:**
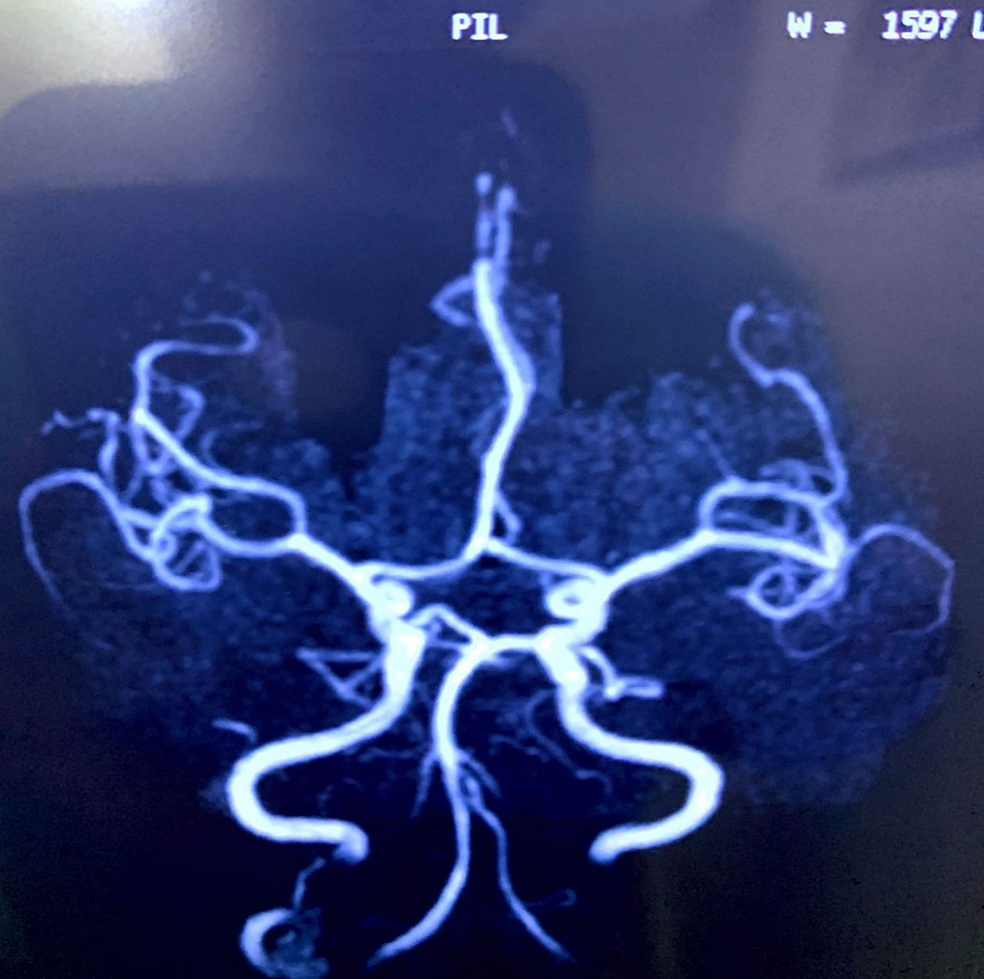
MRA at six-month follow-up demonstrating complete healing of the aneurysm MRA, Magnetic resonance angiogram.

The patient was shifted to the ICU for further observation. The patient received intravenous hypertonic saline, analgesics, and oral nimodipine. A neurological examination 24 hours after the procedure showed no focal neurological deficit. There was a significant improvement in her headache and sensorium in the following days, and the patient was successfully discharged. A magnetic resonance imaging (MRI) scan and magnetic resonance angiogram (MRA) at her six-month follow-up showed complete resolution of the SAH and no detectable aneurysmal sacs (Figure 8).

## Discussion

Fenestration of the basilar artery develops when the paired fetal longitudinal neural arteries fail to fuse by the fifth week of gestation. BAF can occur at any segment of the basilar artery with defective midline fusion. However, it is usually encountered in the proximal segment [6].

The histopathological findings in fenestrations are imperfect medial membrane at both edges of the fenestration, elastin defect, and thinning of the subendothelial layer at the proximal end, resulting in a weakened vessel wall. These intrinsic defects coupled with altered hemodynamics (high shear stress and high-speed turbulence) contribute to aneurysm formation and rupture [5].

Aneurysms are classified based on their size as small (diameter less than 11 mm), large (diameter of 11-25 mm), or giant (diameter greater than 25 mm) [7]. The treatment options for aneurysms include standard coiling, balloon-assisted therapies, stent-assisted therapies, and flow-diverter systems [8]. Vajpeyee et al. in 2013 used a novel double microcatheter-assisted technique for coiling a BAF aneurysm [9].

Surgery of BAF aneurysms is complicated due to the complex geometry of the fenestration, proximity of the lower cranial nerves, difficulty in obtaining adequate surgical exposure, and crowding of arteries in this region [10,11]. In the study by Campos et al., 13 patients had transient lower cranial nerve palsies. One patient had a permanent neurological deficit, and another patient died following surgical treatment [12]. In 2021, a comprehensive review of literature by Korkmaz et al. reaffirmed the notion that ET is superior to a microsurgical approach in terms of higher clinical success rate and lower complication rate [8].

In our patient, each arm of the fenestration sported an aneurysm. The larger left aneurysm had a narrow neck that was ideal for coiling. The smaller right branch aneurysm had a wide neck and was unsuitable for coiling. Therefore, it was flow-diverted with an LVIS stent. The pliability of the material and relatively closed cell design (compared to a conventional stent) made flow diversion achievable. There was a significant cost-cut compared to using a traditional flow diverter.

The coiling of an aneurysm promotes thrombosis by diminishing the blood flow going into the aneurysm, lowering the velocity, prolonging the residence time of blood within the aneurysmal space, and lessening the aneurysmal wall shear stress. Complete aneurysmal healing might take upward of six months [13]. The flow diverter covers the neck of the aneurysm, and its mesh reduces the blood flow into the aneurysm. The stasis of flow within the aneurysm promotes thrombosis. The flow diverter also provides a scaffold for neo-endothelialization across the aneurysm neck [14-16].

## Conclusions

Fenestration of the basilar artery is a rare anatomical anomaly. The endovascular approach is the treatment of choice because of the inherent complexity of these vascular abnormalities. Proper pre-procedural planning and selection of ideal endovascular treatment modalities are crucial for therapeutic success and the best possible clinical outcomes.
